# The common bisulfite-conversion-based techniques to analyze DNA methylation in human cancers

**DOI:** 10.1186/s12935-024-03405-2

**Published:** 2024-07-09

**Authors:** Farhad Jeddi, Elnaz Faghfuri, Sahar Mehranfar, Narges Soozangar

**Affiliations:** 1 Zoonoses Research Center, Ardabil University of Medical Sciences, Ardabil, Iran; 2 Department of Genetics and Pathology, School of Medicine, Ardabil University of Medical Sciences, Ardabil, Iran; 3 Digestive Diseases Research Center, Ardabil University of Medical Sciences, Ardabil, Iran; 4 Department of Genetics and Immunology, Faculty of Medicine, Urmia University of Medical Sciences, Urmia, Iran

**Keywords:** Bisulfite-conversion, Cancer, DNA methylation, Epigenetic

## Abstract

DNA methylation is an important molecular modification that plays a key role in the expression of cancer genes. Evaluation of epigenetic changes, hypomethylation and hypermethylation, in specific genes are applied for cancer diagnosis. Numerous studies have concentrated on describing DNA methylation patterns as biomarkers for cancer diagnosis monitoring and predicting response to cancer therapy. Various techniques for detecting DNA methylation status in cancers are based on sodium bisulfite treatment. According to the application of these methods in research and clinical studies, they have a number of advantages and disadvantages. The current review highlights sodium bisulfite treatment-based techniques, as well as, the advantages, drawbacks, and applications of these methods in the evaluation of human cancers.

## Introduction

DNA methylation is an epigenetic modification that occurs by adding methyl groups to the fifth carbon of cytosine (5mc). DNA methylation has been associated with not only human cancer but also with numerous cellular processes such as embryonic development, transposon inactivation, genomic imprinting, X chromosome inactivation, chromatin structure alteration, and transcriptional repression [1–3]. Methylation modifications are predictive indicators and powerful prognostic markers in the diagnosis and treatment of cancers [4–8].

In this review, we will describe each of the techniques as well as focus on their application in cancer research and diagnosis. Furthermore, the pros and cons of each method will be represented in detail. Not all methods based on sodium bisulfite treatment can be covered in this review; therefore, we will emphasize approaches that are easy to use, readily accessible, and most robust for research centers. In addition, there are several important factors in choosing a technique for DNA methylation study, including the quality and quantity of genomic DNA (gDNA) samples, the specificity and sensitivity required to analyze, the simplicity and strength of the technique, and the accessibility of reagents and specific devices [9].

In this study, we will explain methodologies developed to analyze epigenetic alterations using sodium bisulfite (NaHSO3) treatment platforms, including; MSP (methylation-specific PCR), MS-HRM (methylation-specific high-resolution melting), pyrosequencing, MS-SSCA (methylation-specific single-strand conformation analysis), MS-SNuPE (methylation-specific single nucleotide primer extension), SMART-MSP (sensitive melting analysis after real-time MSP), fast-COLD-MS-PCR, COBRA (combined bisulfite restriction analysis), MS-FLAG (methylation-specific fluorescent amplicon generation), HeavyMethyl, MB-MSP (MutS-based methylation-specific PCR), and NGS-based amplicon sequencing.

## MSP (methylation-specific PCR)

To perform MSP analysis, purified DNA is subjected to alteration with sodium bisulfite. In the sulfonation reaction, all unmethylated cytosine (C) sites change to uracil but the methylated cytosines do not convert [10–12]. In the MSP technique, two pairs of primers, the first pair of primers to methylated sites (M primers) and the second pair of primers to unmethylated sites (U primer) are required for the PCR reaction. To distinguish between unmethylated and methylated DNA, every primer should employ at least one or more CpG dinucleotides [13, 14]. PCR product with M primers is indicative of methylated gDNA and successful PCR reaction with U primer pair is reflective of unmethylated gDNA. MSP can identify a single methylated allele among many unmethylated alleles, making it a susceptible technique in this regard [10].

**Fig. 1:**
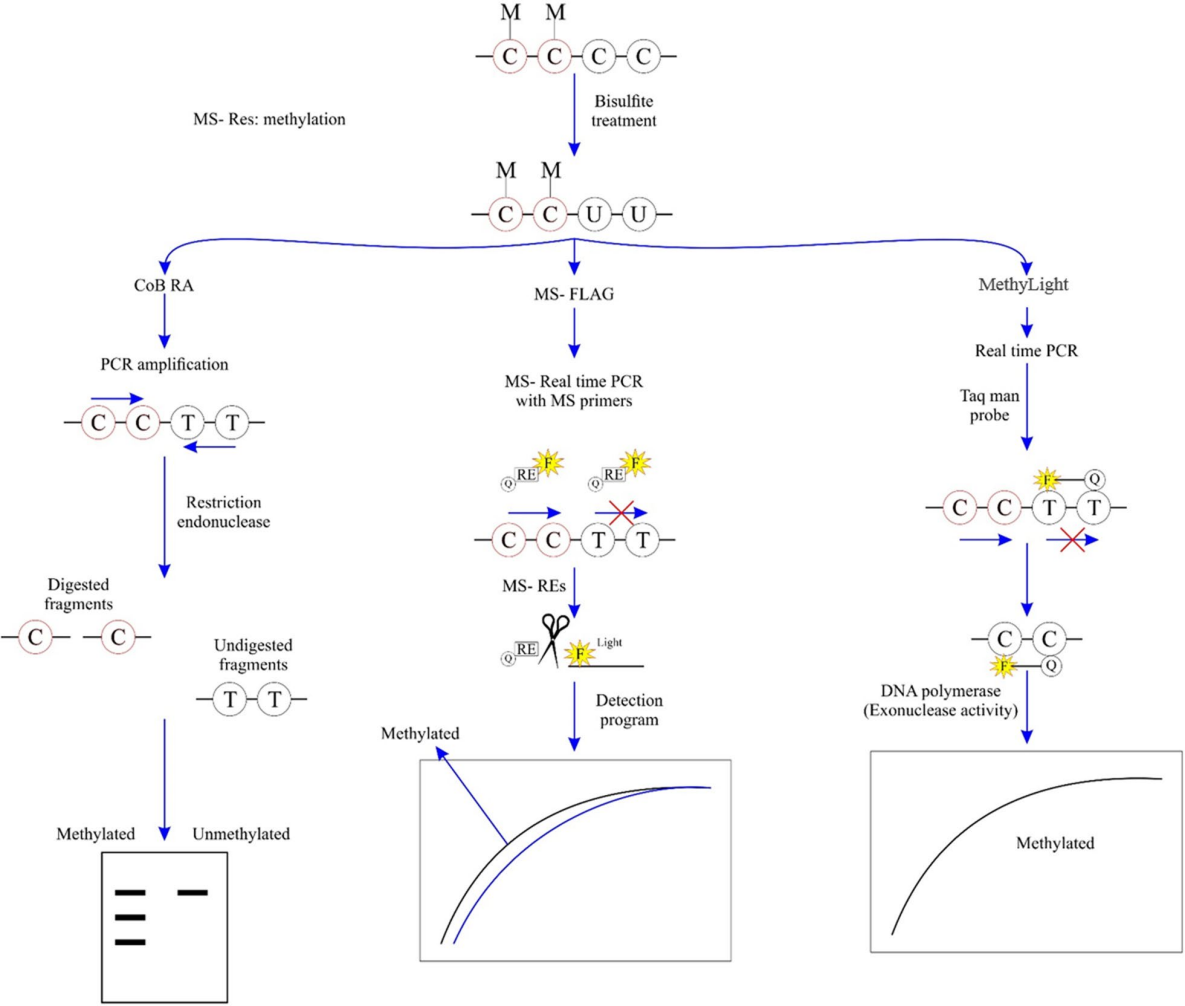
The schematic principle of COBRA: combined bisulfite restriction analysis, MS-FLAG: Methylation-Specific Fluorescent Amplicon Generation, MethyLight. Bisulfite treatment: Bisulfite converts unmethylated cytosine (C) to uracil (U) but methylated (M) cytosine doesn’t change. COBRA: after sodium bisulfite treatment, the PCR process is performed and the PCR products are digested with a specific restriction endonuclease. This enzyme can detect methylated DNA and digest it. The digestion patterns are detected by gel electrophoresis. MS-FLAG: after sodium bisulfite treatment, the real-time PCR is performed with methylated specific primers containing a thermostable endonuclease site. The 5'ends of primers are labeled by a quencher (Q) and a fluorophore (F) and the cleavage sites by Methylation Sensitive Restriction Enzymes (MS-REs) release the quencher from the fluorophore and light to emit. The emitted light depends on the amount of methylated sites in the sample. MethyLight: In this method, the specific methylated primers and the labeled probe (Taq man probe) with a fluorophore (F) and a quencher (Q) detect the methylated DNA. Subsequently, the DNA polymerase cleaves the probe with its exonuclease activity. It causes the fluorescent dye to be released from the quencher and emit light. The emitted light depends on the amount of methylated sites in the target sample

MSP is a qualitative approach for the detection of methylation in individual genes due to its easy experimental design and high sensitivity, capable of detecting one methylated allele among 1000 unmethylated alleles. However, notable drawbacks include its limited specificity, methylation detection solely at primer binding sites, and its ability to detect methylation at only a limited number of cytosine molecules [15]. A different type of MSP, known as MethyLight or QAMA (quantitative analysis of methylated alleles), can quantitatively assess gDNA. In the MethyLight method, MSP is coupled with TaqMan quantitative-PCR reaction, where the fluorescence is utilized to detect the products through the amplification phase. This method can detect one methylated allele among 10,000 unmethylated alleles. Therefore, this method has a higher sensitivity in detection than MSP (Fig. 1) [16–18]. Alternately, Rand, K. et al. have established the ConLight-MSP technique, in which PCR and sulphonation reactions are merged within a TaqMan quantitative-PCR reaction by a further fluorescent probe versus unmodified DNA. An additional probe for unconverted DNA increases the specificity of the PCR process for effective bisulfite-dependent conversion of DNA [19].

Conventional MSP cannot be used in clinical diagnosis for two reasons. Firstly, MSP qualitatively detects the methylation status and secondly, it shows false-positive and false-negative results, particularly when the gDNA is extracted from formalin-fixed, paraffin-embedded (FFPE) tissue samples. However, MethyLight is an appropriate technology for molecular diagnostics [20]. This method has been used to detect epigenetic alterations in several malignancies such as esophageal, breast, ovarian, stem cell, prostate, and colon cancers [21, 22]. One of the important benefits of the MethyLight methodology is its ability to detect a single-copy gene in clinical specimens with low quantities of gDNA [23, 24].

Lee et al. compared the two methods of qualitative MSP and quantitative pyrosequencing to assess the methylation alteration of *MGMT* (O6-methylguanine-DNA methyltransferase), *RASSF1A* (RAS association domain family 1A), *RARb2* (retinoid acid receptor h2) and E-cadherin genes in human salivary gland carcinoma (SGC) samples as well as five cancer cell lines [25]. They found that MSP is a simple and rapid qualitative method for detecting and screening of DNA methylation in tumors. But, the MSP technique was less sensitive than pyrosequencing [25].

Another MSP-based method is McMSP, which evaluates the yields using melting curves [26]. This technique amplifies bisulfited gDNA through specific primers for unmethylated and methylated sites. The ratio of unmethylated and methylated products is obtained quantitatively by comparing the degree of difference between peaks produced in a melting curve [27, 28].

## Pyrosequencing

**Fig. 2:**
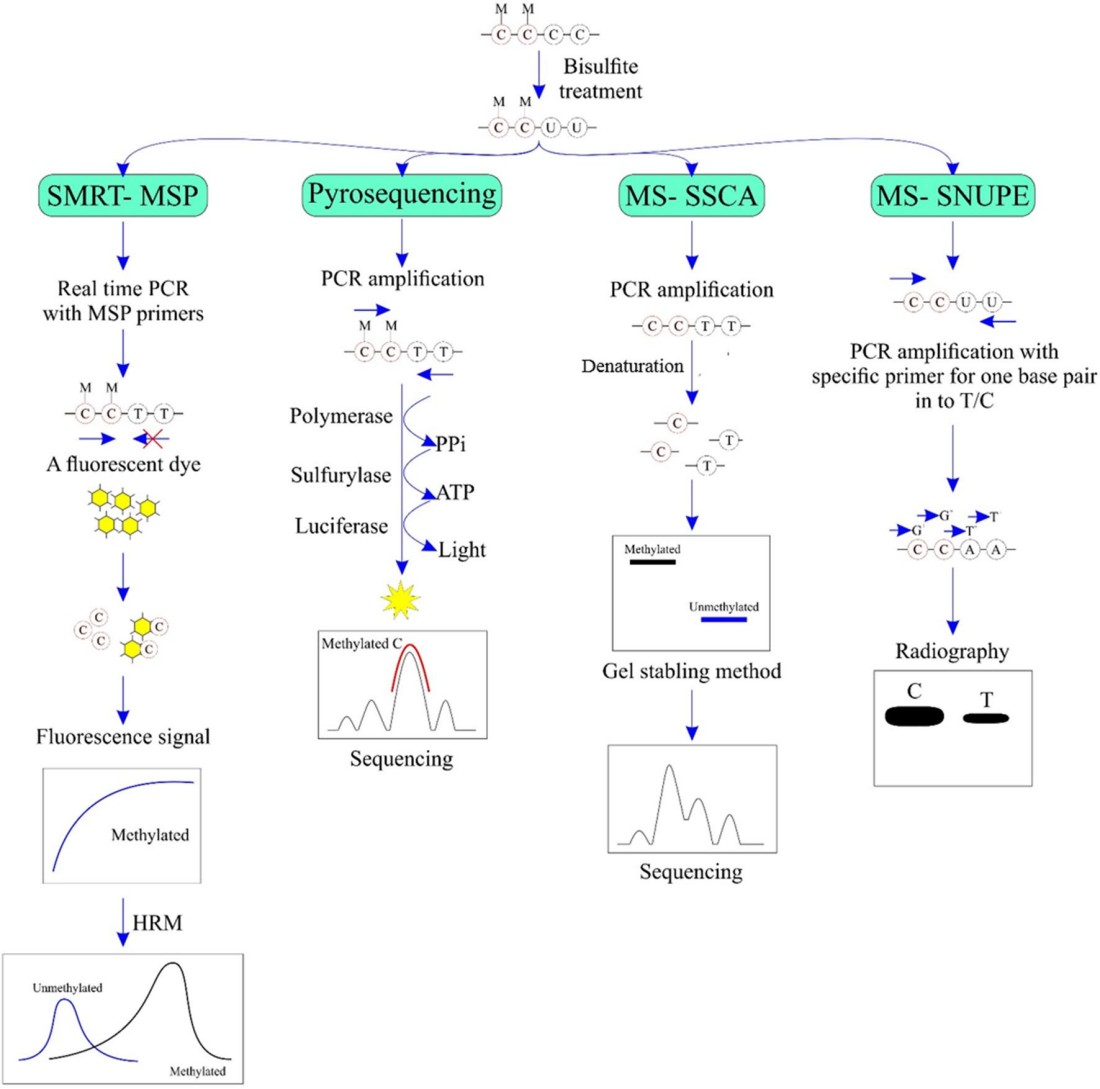
The schematic principle of SMART-MSP: Sensitive Melting Analysis after Real-Time MSP, MS-SSCA: methylation-specific single-strand conformation analysis, MS-SNuPE: methylation-specific single nucleotide primer extension. SMART-MSP: After bisulfite treatment, real-time PCR is performed with methylated specific (MSP) primers with a fluorescent dye. Then, the resulting products are evaluated by HRM (high-resolution melting). Pyrosequencing: After bisulfite treatment and PCR amplification is performed with primer and DNA polymerase. Upon synthesis of the DNA strand, PPi is released and converted to ATP by sulfurylase. ATPs provide the energy for the formation of the luciferase complex, leading to the release of light in an amount proportional to ATP and PPi. MS-SSCA: After bisulfite treatment, the PCR process is performed and the amplified segments are subsequently denatured. Methylation changes are determined by a gel-stabbing method and sequencing. MS-SNuPE: After bisulfite treatment and PCR amplification is performed with a specific primer for one base pair into Cytosine (or Thymine). The proportion of C/T is obtained quantitatively and is separated on radiography

The bisulfite pyrosequencing method is based on bisulfite alteration coupled with PCR reaction [29]. In this technique, the modification of dsDNA (double-stranded DNA) to ssDNA (single-stranded DNA) is facilitated by a biotinylated primer in PCR amplification. Then, ssDNA is annealed to the sequencing primer, followed by the pyrosequencing process. The release of pyrophosphate occurs with the incorporation of a nucleotide in ssDNA, which is evaluated using a luciferase assay. The developed signal is proportional to the quantity of pyrophosphate released. By using this technique, the conversion of C-to-T can be detected and quantified in both rich and poor CpG regions (Fig. 2) [30, 31].

Pyrosequencing technology has several applications including, bacterial strain typing, single nucleotide polymorphism (SNP) genotyping, quantitative detection of CpG island methylation, and detection of mutation in cancers [32–34]. Bakkum-Gamez et al. managed to identify DNA hypermethylation in samples collected from intravaginal tampons by pyrosequencing. For this purpose, they combined a high-throughput procedure with a non-invasive collection technique for early diagnosis of endometrial cancer [35, 36]. The study by Kottaridi et al. has argued that pyrosequencing technology plays an important role as an indicator of malignant tumors [37]. They analyzed the mean methylation of thirteen CpG sites in the *GALR1* promoter in malignant and normal endometrial specimens. In another study, Irahara and coworkers comprehensively measured the long interspersed nuclear element-1 (LINE-1) methylation content in colon cancer through bisulfite pyrosequencing approach, as a beneficial method for research or clinical purposes [32].

## MS-SSCA (methylation-specific single-strand conformation analysis)

MS-SSCA is a technique to study methylated DNA in a specific gene [38]. In this method, genomic DNA is subjected to alteration by sodium bisulfite, then the desired gene is amplified using primers specific for bisulfite-modified sequences. The amplified segments are subsequently denatured and run on a nondenaturing polyacrylamide gel. Strands that differ by as little as a single base substitution can form different conformers and migrate to different positions in the gel (Fig. 2). Nevertheless, there remains a chance that the conformers could comigrate. Therefore, it is recommended to test multiple sets of running conditions [39]. MS-SSCA has been used to determine the methylation status of various gene promoters, including *APC*, *hTERT*, *p16*, *FHIT*, E-cadherin, *hMLH-1*, *RAR-b2*, *RASSF1A* and *TIMP-3* in esophageal adenocarcinoma [40]. However, due to the relatively low sensitivity of MS-SSCA analysis, researchers may have also missed the detection of small populations of cells with a partially methylated allele [41].

## MS-HRM (methylation-specific high-resolution melting)

Inthe MS-HRM system, the melting curves of unidentified samples and PCR products obtained from unmethylated and methylated DNA standards are compared. The melting profile of the methylated allele is different from the unmethylated allele of the same locus because both alleles are completely different in terms of GC content. Therefore, to explore the methylation pattern of a new locus, the melting profiles of methylated and unmethylated controls have to be compared with the profile of PCR products originating from that locus [42, 43]. The two crucial advancements that have enabled this technique are the identification of dyes that bind to double-stranded DNA without impeding PCR amplification and the introduction of highly sensitive instrumentation capable of detecting even the slightest change in fluorescence intensity [21]. Recently, some methodologies have been developed to calculate the methylation levels using HRM curves [44]. In addition, MS-HRM is a sensitive technique for detecting low methylation levels, which are important in cancer samples that may have small amounts of methylated sequences because of tumor heterogeneity or the presence of normal tissues [45]. Spitzwieser and colleagues applied the MS-HRM method for detecting the methylated sites in *MGMT*, *GSTP1*, *DAPK1*, *RASSF1A*, *CCND2,* and *HIN-1* promoters in breast cancer. In this study, they chose the MS-HRM technique because they aimed at characterizing small alterations in the methylation of gene promoters and evaluating a DNA molecule with a low level of methylation [46]. In another study, MS-HRM is used to analyze promoter methylation status in 7genes, *RASSF1A, RUNX3, MGMT, CBLN4, SFRP1*, *SFRP2* and *INA*, in brain tumor. This methodology has been proved useful for characterizing or diagnosing brain glioma tumor [47].

## SMART-MSP (sensitive melting analysis after real-time MSP)

SMART-MSP is a combination of HRM and real-time MSP technology. After changing gDNA using sodium bisulfite, the real-time amplification is performed with a fluorescent dye and methylated specific primers. To obtain the quantitative data of real-time MSP through CT values, a control assay is run for each analysis. Also, unmethylated and methylated DNA standards are used to detect true positive results. Then, the resulting products are evaluated by HRM. The melting peaks of methylated standards and test samples are compared to detect true positives (Fig. 2) [28]. Significantly, HRM analysis may be specifically appropriate for identifying false-positive outcomes, rather than for scenarios where incomplete conversion results in a slight overestimation of methylation levels. This is due to the fact that the signal intensity from methylated and fully converted molecules will be considerably higher in such instances compared to the signal obtained from incompletely converted molecules [48]. SMART-MSP and MS-HRM methods were successfully applied to determine the methylation level of the *RARB* and *CDKN2A* (p16) promoters in non-small cell lung cancer (NSCLC) samples [49].

## MS-SNuPE (methylation-specific single nucleotide primer extension)

MS-SNuPE technology is based on a custom genotyping system, namely SNuPE, which was initially designed for studying SNPs [50]. In this method, gDNA is converted by bisulfite, and specific primers are designed to anneal to the sequence up to the base pair exactly before the interested CpG. The specific primer is permitted to amplify one base pair into cytosine (or thymine) and the proportion of C/T is obtained quantitatively. Initially,the radioactive ddNTPs are applied as an indicator of the primer extension. MS-SNuPE can be coupled with other methods such as bisulfite pyrosequencing and fluorescence-based techniques (Fig. 2) [51–54].

El-Maarri et al. modifiedthis procedure, in which the products are separated by dHPLC (denaturant HPLC), and non-radioactive ddNTPs are applied instead of radioactive ddNTPs. Combined SNuPE and SIRPH (SNuPE ion pair reversed-phase HPLC) is a quantitative assay in which several sites can be evaluated simultaneously [55]. Furthermore, MS-SNuPE has been used to quantify and detect aberrant methylation in cancers when small amounts of DNA are available [21]. This method is labor-intensive and has the drawback of necessitating the use of radioactive materials. Alternatively, the SNaPshot technology from Applied Biosystems can serve as a detection platform, eliminating the need for radioactive labeling [48].

## COLD-MS-PCR (co-amplification at lower denaturation temperature MS-PCR)

In this technique, gDNA is first converted by sodium bisulfite. Then, PCR process is performed on converted DNA through CpG-free primers. COLD-PCR relies on the low denaturation temperature; therefore, unmethylated fragments are preferentially amplified. The resulting amplicons can be detected via an intercalating color and differential melting analysis, followed by sequencing of the resulting fragments [56]. COLD-MS-PCR is capable of detecting traces of unmethylated sequences among the population of methylated copies. Therefore, a COLD-PCR assay is highly sensitive for detecting hypomethylated and unmethylated DNA in various cancers [57–61]. On the other hand, COLD-PCR techniques, such as Fast-COLD-MS-PCR, E-ice- COLD-PCR (enhanced-improved and complete enrichment), enrich rare mutations using an optimal Tc (critical temperature) in the PCR reaction [62]. Fast-COLD-MS-PCR is a highly specific, sensitive, rapid, inexpensive, and easy-to-implement technique. E-ice-COLD-PCR can be applied to enrich methylated DNA, which can detect a single nucleotide by pyrosequencing. In this method, the blocker probes were used to stop the amplification of unmethylated DNA. The e-ice-COLD-PCR method can identify the presence of low-methylated regions, works with low amounts of starting materials, and can be performed at a short time with high throughput [63].

A wide range of clinical samples can be studied using the COLD-PCR system such as circulating tumor cells, frozen tumors, and FFPE samples. The main limitation of this technique for clinical diagnosis is the difficulty of assaying several samples in the same reaction [64].

### COBRA (combined bisulfite restriction analysis)

COBRA is a quantitative technique for analyzing DNA methylation at aspecific genome locus. The gDNA is treated with sodium bisulfite, then, the treated gDNA is amplified using PCR and the resulting amplicons are digested with restriction endonuclease [65]. The specific restriction enzyme can detect methylated DNA and digest it. The digestion patterns are detected by gel electrophoresis and densitometry. This technique is more time-consuming than the MSP method because it has an extra enzyme digestion step (Fig. 1) [66]. COBRA has been extensively applied in epigenetics studies such as DNA methylation changes in cancer research [67–70], detecting methylation status of the genome through developmental processes in mammals [71, 72], and characterizing altered methylation patterns of imprinted genes [73]. Researchers combined the COBRA technique with DHPLC assay (denaturing high-performance liquid chromatography) to detect *H19* hypomethylation in Russell-Silver syndrome and Alu hypomethylation in gastric cancer [74, 75].

In another study, Lim et al.used COBRA and bisulfite modification sequencing analysis to demonstrate that hypermethylation of X Antigen Family Member 1A (*XAGE-1*) promoter can silence the expression of *XAGE-1* mRNA [76]. In acute lymphocytic leukemia (ALL), COBRA and MSP have been successfully used to compare the methylation pattern of five genes in patients with initial presentation and relapse. The results of both techniques showed that DNA methylation status at relapse remained stable in 88% of patients for estrogen receptor (ER), 72% for multidrug resistance gene 1 (*MDR1*), 60% for p15, 80% for p16 and 92% for p73 [77]. Also, COBRA and MSP are applied to evaluate the methylation of p15 and *MGMT* genes in ALL and AML patients. In this study, the methylated promoter of p15 is more frequent in AML samples than in ALL samples. On the other hand, the methylation of the *MGMT* is found in 35.59% of acute leukemia samples and there was no significant difference between the two groups of patients [78].

In another study, Aparicio et al. compared four methods, MethyLight, COBRA, MS-SNuPE, and pyrosequencing, to analyze the methylation of the melanoma-associated antigen 1 (*MAGE-A1*) and *LINE-1* promoters in HCT116 (colon cancer cells) and T24 (bladder cancer cells). In this study, pyrosequencing methodology had the best reproducibility and the highest signal-to-noise ratioin detecting methylation changes in the *MAGE-A1* and *LINE-1* promoters [79], and it was shown that the MethyLigh technique can determine the percentage of methylated CpGs in all the amplified sequences [79].

The methylation status of the *PTEN* tumor suppressor gene and *PTENP1* pseudogene in endometrial cancer was assessed by the COBRA method. It was found that *PTEN* was not methylated*,* whereas a 5′-terminal region of pseudogene *PTENP1* was methylated. Therefore, these findings point to the relation between suppression of pseudogene *PTENP1* by methylation and the pathogenicity in endometrial cancer [80]*.* Recently, the methylation status in the *RASSF1* promotor was analyzed by modified COBRA protocols with the Microfluidics Electrophoresis (LabChip) for accessible and rapid breast cancer screening. In this study, the PCR conditions were optimized to obtain a high throughput product by designing the primers for two distinct CpG sites of interest. Lastly, improvements in the enzyme digestion step and using sensitivity analytical method led to the bypass of the post-PCR concentration and purification in COBRA assays [81].

COBRA is a low-throughput technique that is limited to analyzing CpGs found within enzymatic restriction sites. Additionally, the method is somewhat labor-intensive but cost-effective [22]. An enhanced protocol for COBRA, known as Bio-COBRA, has been created utilizing a microfluidic platform to enable more high-throughput, precise, and quantitative DNA methylation analysis [82].

## MS-FLAG (methylation-specific fluorescent amplicon generation)

MS-FLAG is a high-throughput, quantitative MSP method for detecting and screening DNA methylation. Furthermore, this method can be used for DNA diagnostics [83]. In MS-FLAG, the MSP primers are cleaved using a thermostable endonuclease such as PspGI. The 5'ends of primers are labeled by a quencher and a fluorophore and the cleavage sites create the fluorescence signal in real time (Fig. 1) [83]. Similar to other MSP-based techniques, the MS-FLAG method is sensitive but SMART-MSP is more cost-effective than MS-FLAG because the latter needs a thermostable endonuclease and fluorescently labeled primers. Multiplex MS-FLAG analyses can be performed to reduce workload and costs due to the use of differently labeled primers [48, 83]. Bonannoet al. implemented MS-FLAG and MSP techniques to detect methylation profiles of three gene promoters insurgical samples of lung adenocarcinoma. The results obtained by MS-FLAG indicated the hypermethylation of *CDKN2A* (*p16*), *GATA5*, and *RASSF1* at the levels of 47%, 85%, and 57%, respectively. On the other hand, the results of conventional MSP were consistent with those of MS-FLAG [83].

## HeavyMethyl

**Fig. 3:**
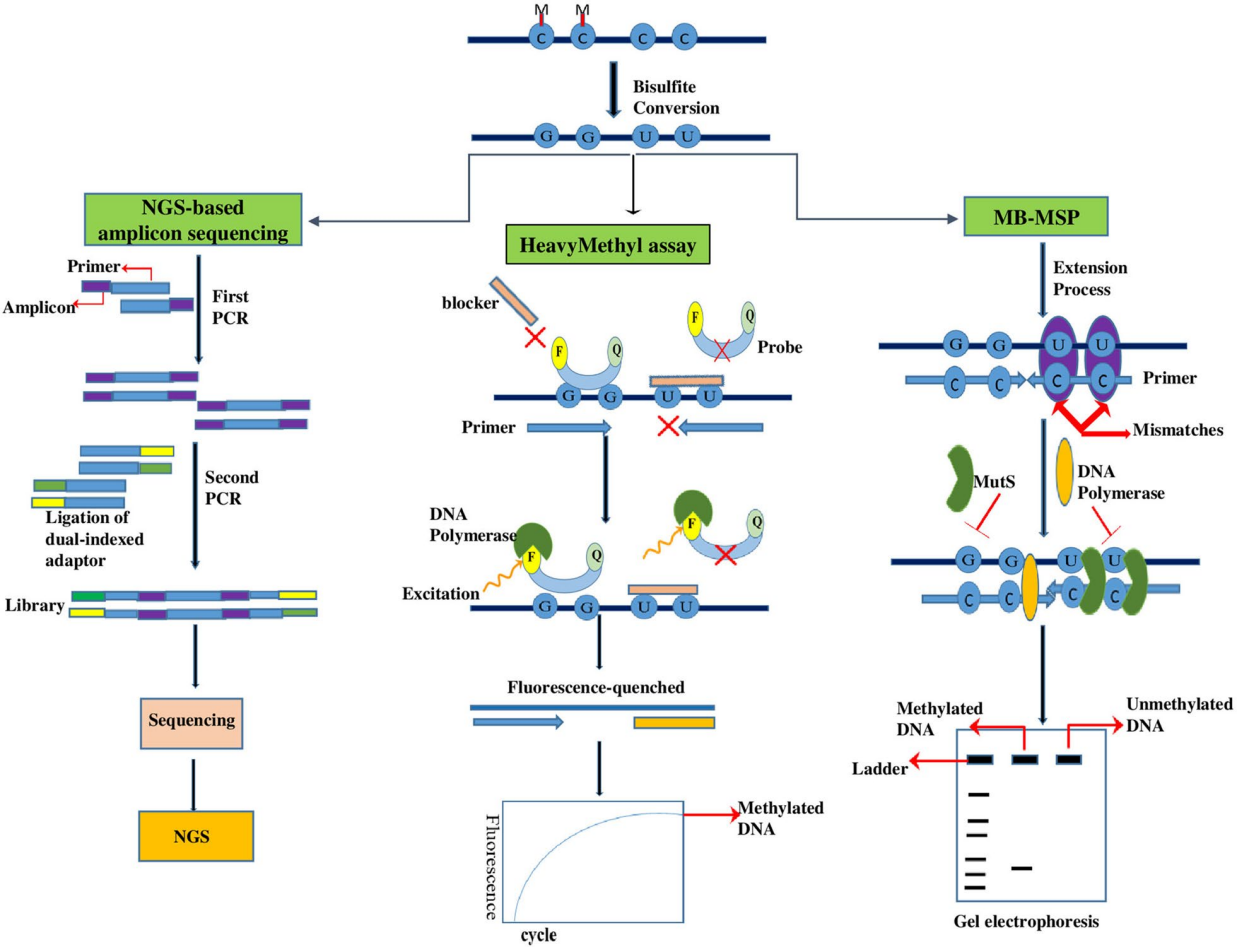
The schematic principle of NGS-based amplicon sequencing: Next-Generation Sequencing (NGS)-based amplicon sequencing, HeavyMethyl assay, MB-MSP: MutS-based methylation-specific PCR. Bisulfite conversion: Bisulfite converts unmethylated cytosine (C) to uracil (U) but methylated (M) cytosine doesn’t change. NGS-based amplicon sequencing: after bisulfite conversion, the PCR process is performed twice. In thefirstPCR, the gene-specific primer carries the amplicons for the second PCR. In the second PCR, the products are reamplified with specific adaptor primers attached to the amplicon sequences. Afterward, the appropriate library can be applied for sequencing and NGS analysis, HeavyMethyl assay: after bisulfite conversion, the blocker oligonucleotides bind to unmethylated sites and block primer binding sites. Alternatively, the blocker cannot hybridize to methylated DNA sites, allowing the primers to bind to their binding sites and amplify the target. The labeled probe with a fluorophore (F) and a quencher (Q) detects the PCR products and hybridizes with them. Subsequently, the DNA polymerase cleaves the probe with its exonuclease activity. It causes the fluorescent dye to be released from the quencher and emit light. The emitted light depends on the amount of methylated sites in the target sample. MB-MSP: after bisulfite conversion, and PCR amplification, the MutS protein binds to the mismatch site (G/U) of the unmethylated conversion and primer complex. The binding of MutS prevents DNA polymerase to this complex, so inhibiting the replication of unmethylated DNA. Finally, gel electrophoresis analysis is applied to detect methylated and unmethylated fragments

HeavyMethyl assay is based on detecting methylated and unmethylated DNA using blockers combined with methylation-unspecific primers. After bisulfite-treated DNA, these primers have been designed to bind next to the CpG-rich regions, for which oligonucleotide blockers are designed to bind only to unmethylated DNA. Thus, the blocker oligonucleotides cannot hybridize to methylated DNA sites, allowing the primers to bind to their binding sites and amplify the target. Therefore the blockers can bind to unmethylated targets, and block primer binding sites. The labeled probe with a fluorescent dye and quencher detects the PCR products. MutS protein can bind to the mismatch site (G/U) of the unmethylated conversion and primer complex in the following PCR amplification. Subsequently the correct hybridization of primers and the probe, the DNA polymerase enzyme cleaves the probe with its exonuclease activity. It causes the fluorescent dye to be released from the quencher and emit light. The emitted light spectrum depends on the amount of target methylated DNA molecules, which allows accurate determination of methylation levels (Fig. 3) [48].

Kneip et al. used the HeavyMethyl method for analyzing SHOX2 DNA methylation in patients with lung cancer. They found that DNA methylation of *SHOX2* is a highly sensitive and specific biomarker for analyzing lung cancer patients. Sensitivity and specificity are slightly higher when using bronchial aspirates when compared with blood plasma [84]. The results of another study to measure DNA methylation of *SHOX2* in lung cancer with HeavyMethyl technology indicated that this test is a reliable and powerful tool for lung cancer diagnosis [85, 86]. In another study, *ALX4* and *SEPT9* gene methylation were analyzed by HeavyMethyl assay and they were introduced as potential biomarkers to identify colorectal cancer [87].

## MB-MSP (MutS-based methylation-specific PCR)

MB-MSP method is a combination of MutS and MSP technology. The MutS is a key protein in mismatch-specific recognition that can identify methylated guanine-containing base pairs and bind to locations between normal and methylated bases [88].

Therefore, developing a method based on the MutS protein led to an increase in sensitivity and specificity of methylation detection [89]. This method has the advantages of high sensitivity and specificity, speed, simplicity, and broad applicability. In this technique, after modifyingthe gDNA sample using sodium bisulfite, PCR amplification is performed with two pairs of specific primers for unmethylated and methylated DNA. The MutS protein can bind to the mismatch site (G/U) of the unmethylated conversion and primer complex in the following PCR amplification. The binding of MutS prevents DNA polymerase to this complex, so inhibiting the replication of unmethylated DNA. Therefore, this protein can inhibit not only non-specific primer amplification but also the replication of unmethylated DNA. Finally, gel electrophoresis analysis is applied to detect methylated and unmethylated fragments (Fig. 3) [90]. The study by Zhang et al. has argued that MB-MSP technology is an improved method for detecting DNA methylation statute, has potential application value, and predicts early cancer diagnosis. They evaluated the methylation analysis of *CLEC11A*, *SEPT9*, and *ACP1* genes in liver cancer samples by the MB-MSP method, with a 1.5 h detection time and 0.5% detection limit [90].

## NGS-based amplicon sequencing (Next generation sequencing-based amplicon sequencing)

The amplicon-based bisulfite gDNA methylation method combines bisulfite conversion of gDNA with amplicons (targeted amplification of the regions of interest), the sequencing library, and next-generation sequencing (NGS). This method provides quantitative DNA methylation with genome-wide analysis and single-base pair resolution [91]. At the start of the workflow, gDNA is treated with sodium bisulfite, then the converted DNA is amplified using the PCR process twice. In the first PCR, the gene-specific primer carries the amplicons for the second PCR. In the second PCR, the products are reamplified with specific adaptor primers attached to the amplicon sequences. Afterward, the appropriate library can be applied for sequencing and following data analysis (Fig. 3) [92, 93].

Recently, da Silva et al. assessed MLH1 promoter methylation in colorectal cancer with an NGS-based amplicon sequencing method. They observed above 10% *MLH1* methylation in cancer samples with MLH1/PMS2 loss. In these samples, the MLH1 methylation was highly associated with the *BRAF* mutation [94]. This study showed that NGS-based amplicon sequencing has great specificity and sensitivity to detect *MLH1* methylation in CRC patients.

## Conclusion

DNA methylation analysis has provided a comprehensive overview of the relationship between human cancer and epigenetic changes. The functional effect of aberrant DNA methylation and the extensive changes in DNA methylation in cancer development have led to the expansion of various techniques to describe methylation patterns. Further understanding of the association between the effects of DNA methylation at the molecular level and its clinical relevance may pave the way for future advances in the surgical and pharmacological management of malignancies. This paper focuses on several sodium bisulfite treatment-based techniques for DNA methylation studies. The methodologies studied in this article have different drawbacks and advantages that should be assessed before beginning methylation analysis. Comparisons of sodium bisulfite treatment-based techniques are presented in Table 1.

**Table 1:** Comparison of selected characteristics between bisulfite-conversion-based techniques

Methods	Advantages	Drawbacks	Analytical sensitivity^a^	Input bisulfite-converted DNA	LOD^b^	Quantitative accuracy	Type of cancer	Reference
MSP	∙ Cost effective ∙ A rapid assessment methodology ∙ Suitable for FFPE tissues ∙ Simple protocol	∙ False positives ∙ Low quantitative accuracy ∙ Only qualitative ∙ Requires two different pairs of primers ∙ PCR contamination	High	∼ 100 ng/µl	0.1	Low	Esophageal, breast, ovarian, prostate, bladder, lung, gastric and colon cancers	[10, 15, 21]
MethyLight	∙ Not much work needed ∙ High throughput ∙ High Quantitative ∙ High Sensitive	∙ Expensive ∙ Complexity of the assay ∙ A control assay is required	High	< 20 ng/ µl	0.01	High	Squamous cell carcinoma, head and neck gastric, breast, cervical, and colorectal cancers	[15, 95]
Pyrosequencing	∙ Obtaining quantitative results on distinct CpG sites ∙ High quantitative accuracy ∙ Automatic analysis of data ∙ Helpful for genomic imprinting study ∙ Applicable for evaluating bisulfite-converted DNA without using MSP	∙ Digital PCR or cloning procedures are required if a single fragment is to be sequenced ∙ Development of many secondary constructions (due to low-temperature reaction)	Medium	25–100 ng/µl	2	High	Colon, gastric, bladder, breast, lung, head and neck, and endometrial cancer	[15, 30, 30, 36, 51, 96–98]
MS-SSCA	∙ Identifying single base modifications in a section ∙ Several samples can be screened for methylation ∙ Quick genomic sequencing	∙ Semi-quantitative ∙ If only a single nucleotide difference is present, the sensitivity of the method is low	Low	∼25 ng /µl	–	Medium	Esophageal adenocarcinoma, breast, and colorectal cancers	[38, 39, 41]
MS-HRM	∙ No PCR bias ∙ High quantitative accuracy ∙ High sensitivity ∙ High-throughput ∙ Low risk of PCR contamination (closed-tube assay) ∙ Increased analytical sensitivity	∙ If excessive CpG sites are present between the primers, analysis can be difficult ∙ Requires special equipment ∙ The use of fluorescent dyes	High	∼ 20 ng/µl	1	Medium	Breast, colorectal, bladder, cervical cancers, and glioma tumor	[22, 45, 99–101]
SMART-MSP	∙ Low false-positive rate ∙ High-throughput ∙ Low risk of PCR contamination (closed-tube assay)	∙ Needs a control assay ∙ The use of fluorescent dyes ∙ Requires special equipment	High	25 ng/µl	0.1	High	Lung, breast, ovarian cancers, and malignant pleural mesothelioma	[28, 49, 102, 103]
MS- SNuPE	∙ Capable of analyzing several CpGs sites simultaneously ∙ Does not use restriction enzymes	∙ Requires radioactive material	Medium	50–200 ng/µl	0.1	High	Breast and bladder cancers	[52, 54, 54, 104]
Fast-COLD-MS-PCR	∙ Applicable for detecting sequences with low CpG density ∙ High sensitive technique for diagnostic purposes ∙ Capable of detecting low-density unmethylated CpG sites when combined with HRM or Sanger sequencing ∙ Highly flexible Tc	∙ Needs CpG-free primers on bisulfite-converted DNA	High	2 ng (corresponding to ∼700 DNA molecules)	–	High	Glioma, breast, lung, kidney, and colon cancers	[56, 62, 63]
COBRA	∙ Cost-effective ∙ High quantitative accuracy	∙ Low throughput ∙ Limited to digestion sites with restriction enzymes	Medium	9.1–37.5 ng/ µl	3	High	Breast, head, and neck cancers, hepatocellular and squamous cell carcinoma	[61, 65, 81]
MS-FLAG	∙ Multiplexing is possible ∙ High quantitative accuracy ∙ Low false-positive results	∙ Requires gel electrophoresis ∙ Low resolution of gels	High	∼ 5 ng/µl	0.01	High	Lung	[83]
HeavyMethyl	∙ Low false-positive results ∙ Requires the least amount of DNA	∙ Needs blocker oligonucleotides ∙ Needs extensive optimization	High	30 pg/µl	0.1	High	Lung, colorectal, and prostate cancers	[86, 105–107]
MB-MSP	∙ Simplicity ∙ Speed ∙ High specificity, sensitivity ∙ Broad applicability	∙ Not available	High	10 ng/ µl	0.5	High	Liver cancer	[90]
NGS-based amplicon sequencing	∙ High quantitative with genome-wide coverage ∙ High throughput ∙ Resolution at the single base with high speed ∙ Less input DNA	∙ Hard to analyze in repetitive sequences ∙ The huge amount of data in the form of short reads ∙ Clonal DNA amplification ∙ Expensive equipment and technical support	High	1 ng/µl	–	High	Colorectal cancer	[91, 92]

^a^Sensitivity parameter depends on the specific assay and factors such as input DNA concentration, quality, and PCR process conditions. For this reason, we have not characterized particular values for this factor

^b^Ratio of methylated cytosine to unmethylated cytosine (%)

MSP: methylation-specific PCR, MS-SSCA: Methylation-specific single-strand conformation analysis, MS-HRM: Methylation-specific high-resolution melting, SMART-MSP: Sensitive Melting Analysis after Real-Time MSP, MS-SNuPE: Methylation-specific single nucleotide primer extension, Fast-COLD-MS-PCR: Fast co-amplification at lower denaturation temperature PCR, COBRA: combined bisulfite restriction analysis, MS-FLAG: Methylation-Specific Fluorescent Amplicon Generation, MB-MSP: MutS-based methylation-specific PCR, NGS-based amplicon sequencing: Next-Generation Sequencing (NGS)-based amplicon sequencing

## Data Availability

No datasets were generated or analysed during the current study.
